# Mental health outcomes and quality of life of Ukrainian refugees in Germany

**DOI:** 10.1186/s12955-023-02101-5

**Published:** 2023-03-09

**Authors:** Johanna Buchcik, Viktoriia Kovach, Adekunle Adedeji

**Affiliations:** 1 Department of Health Sciences, Faculty of Life Sciences, University of Applied Sciences, Hamburg, Germany; 2 Bremen International Graduate School of Social Sciences (BIGSSS), Constructor University, Bremen, Germany

**Keywords:** Mental health, Depression, Anxiety, Quality of life, Refugee, War in Ukraine

## Abstract

The war in Ukraine has generated an increase in the number of refugees. As one of the top recipients of refugees, Germany has introduced policies to ease the integration of Ukrainians. The current study explores mental health outcomes and their association with quality of life among a sample of Ukrainian refugees in Germany. Cross-sectional data were collected from a sample of Ukrainian refugees in Germany (n = 304) using standardised instruments. A t-test was used to check for possible significant differences based on gender. Multiple regression analysis was used to analyse potential associations between general health (GHQ-12) and depressive symptoms and anxiety (PHQ-4), and quality of life (EUROHIS-QOL 8 item). Female participants reported significantly higher psychological distress, depressive symptoms and anxiety. The significant model (*p* < .001) for the males accounts for 33.6% of the variance in quality of life. General psychological distress (β = − .240) and depressive symptoms and anxiety (β = − .411) are associated with decreased quality of life. For the female sample (*p* < .001), the model explains 35.7% of the variance in quality of life. General psychological distress (β = − .402) and depressive symptoms and anxiety (β = − .261) are associated with decreased quality of life. The current study provides the first knowledge on the prevalence of mental health problems and their associations with quality of life among Ukrainian refugees. The findings further identify the vulnerability of women refugees to poorer mental health outcomes. The results also confirm that traumatic experiences in the context of war explain a considerable bulk of mental health problems.

## Introduction

The global number of displaced persons has continued to increase over the last decade. This increase is ascribed to persecution, conflicts, violence, and human rights violations in several regions and countries worldwide. According to the UN Refugee Agency, nearly 89.3 million people were displaced forcibly in 2021, compared to 33.9 million in 1997. Germany, as one of the top recipients of refugees, recorded about 1.3 million refugees at the end of 2021, mostly from Syria, Afghanistan and Iraq [1]. The war in Ukraine since February 2022 causes a significant increase in this number, with about 610.100 registered refugees from Ukraine between February and April 2022 [2].

Although social and political discussions on integrating and providing care for refugees continue, research shows that fleeing war, persecution, or conflicts correlate with long-term mental health problems [3–5]. Peconga and Høgh Thøgersen [6] found in a sample of 8176 that refugees are at considerable risk of developing post-traumatic stress, mental health problems and disorders compared to the general population without experiences of displacement. Similarly, Kirmayer et al. [7] suggest that refugees are often exposed to stressors before (pre-migration stressors), during (migration-related stressors) and after arriving in the host country (post-migration stressors and acculturation-related stressors). These stressors may lead to higher risks and exposures, promoting psychological distress and disorders [7].

A systematic review and meta-analysis, including 37 publications, shows that prevalence rates are higher for refugees than for labour migrants regarding depression (44% vs 20%) and anxiety (40% vs 21%) [8]. Furthermore, Steel et al. [9] show a wide variance in prevalence rates. A total of 161 articles comprising 81.866 refugees and other conflict-affected persons from 40 countries reveal rates of depression with high variability (3–85.5%). These variances confirm that fleeing conflicts and other human rights violations might lead to more psychopathological symptoms [10, 11]. Findings from these studies agree that psychological stress among refugees is often revealed as general psychological distress, anxiety, depression and post-traumatic stress disorder (PTSD) [6, 7, 12]. Furthermore, the prevalence and intensity of psychological distress are assumed to vary based on various demographic characteristics, such as age and gender. For example, Tekin et al. found that female refugees reported more psychological distress [13]. Females are confronted with a more difficult migration experience and face more significant challenges integrating into the host community [14].

Furthermore, research on the quality of life shows that the mental health of refugees plays a significant role in their quality of life outcome. A systematic review of 15 articles found that lower scores in the quality of life correlate with traumatic and stressful events experienced and can directly impact refugees' psychological health [15].

While researchers have extensively explored mental health and its association with quality of life outcomes among different refugee groups, the uniqueness of the Ukrainian refugee migrational experience is expected to present new insight into refugees' psychological health and quality of life outcomes. The political support and social development in Germany regarding the conflict in Ukraine are assumed to have impacted the transition experience of many Ukrainian refugees in Germany and many other European states. The German government's evident solidarity with the Ukrainian people allows for quick integration through legislation supporting, for example, automatic residence permits and providing Ukrainian refugees access to the labour market and a stipend for social support, as seen by the federal government's central support [16]. Similarly, the community's social acceptance and collective approval displayed by the German community arguably further make the Ukrainian refugee migration experience unique from other refugee groups.

These experiences and characteristics of the Ukrainian refugees and their potential consequences on their quality of life and mental health outcomes remain unexplored. The current study proposes an exploration of the mental health of Ukrainian refugees living in Germany and its association with quality of life. The following objectives were set:

1. To explore the prevalence of psychological distress among Ukrainian refugees in Germany.
2. To investigate gender differences in the quality of life and mental health outcomes among Ukrainian refugees in Germany.
3. To explore mental health outcomes as predictors of quality of life among Ukrainian refugees in Germany.

## Methods

Quantitative data on mental health outcomes, quality of life, socioeconomic status (SES) and demographic characteristics were collected among a Ukrainian refugee sample in Germany. Participants in this study were women and men aged 18 and above who had a primary residence in Ukraine before the war started and sought refuge in Germany. 389 Ukrainian refugees attempted the online and face-to-face questionnaire between May and August 2022. The LimeSurvey Platform was used for online data collection. Cases with extensive missing data (n = 85) were removed from the dataset. Data from 304 participants were included in the current analysis.

### Procedure

The study used a cross-sectional design with standardised questionnaires. Compliance with ethical standards was assessed and approved by the ethical committee of the Competence Center Health of the Hamburg University of Applied Sciences (Approval from 21.03.2022). Participants were required to consent to study participation. The recruitment of participants included active and passive steps. Active steps were undertaken by trained interviewers speaking Ukraine. The interviewer approached participants in public places, registration offices for first admittance, various other meeting points for Ukraine refugees (e.g. churches) and public streets near job centres. In these places, subjects were invited to complete the paper and pencil questionnaire. As passive recruitment, information sheets in the Ukraine language—including study descriptions and a QR-Code and link to the online questionnaire—were distributed and posted in public areas, stores, and social media.

### Measures

The study questionnaire was provided in the Ukrainian language. We followed the established back-translation procedure using the standardised German version of the survey instruments. The first translator, fluent in Ukrainian and German, translated the original questionnaire into Ukrainian. Another translator independently back-translated the Ukrainian survey into the German language. We pilot-tested the survey with Ukrainians to receive feedback on the clarity of the questions. Furthermore, Cronbach’s alphas score was satisfactory for all scales (i.e. PHQ-4, α = 0.81; PHQ-2, α = 0.70; GAD-2, α = 0.78; GHQ-12, α = 0.83; EUROHIS-QOL, α = 0.78).

#### Mental health outcomes

Self-reported measures were applied to assess the severity of mental health outcomes. These included the 12-item General Health Questionnaire (GHQ-12; [17]), which collects data on participants' mental health status within the last weeks. It includes questions on a four-point Likert Scale for responses, e.g. about feeling scared, feeling under pressure and being able to concentrate. Sum scores for these items range between 0 and 36. Higher scores indicate a greater degree of psychological distress. The GHQ-12 presented good reliability in the current sample with a Cronbach's alpha value of 0.83. For descriptive analysis, GHQ-12 sum scores were categorised. Scores ranging from 0 through 12 were coded as "normal range", scores 13 through 15 as "mild psychological distress", scores 16 through 19 as "psychological distress", and scores from 20 through 36 were coded as "severe psychological distress". Furthermore, the Patient Health Questionnaire-4 (PHQ-4; [18]) is an ultra-brief questionnaire that consists of a 2-item depression scale (PHQ-2) and a 2-item anxiety scale (GAD-2). It includes questions about feeling joyful, sad, hopeless, nervous, anxious and worried. Sum score range between 0 and 12 [18]. The PHQ-4 presented good reliability in the current sample with a Cronbach's alpha value of 0.81. Furthermore, the aggregate score for depressive symptoms and anxiety were computed separately. For depressive symptoms (PHQ-2), a score between 0 and 6 was achievable. The PHQ-2 presented good reliability in the current sample with a Cronbach's alpha value of 0.70. Similarly, a total anxiety score (GAD-2) ranged between 0 and 6. The questionnaire presented good reliability in the current sample with a Cronbach's alpha value of 0.78. For descriptive analysis, PHQ-4 scores from 0 to 2 were coded as "normal", 3 to 5 as "mild", 6 to 8 as "moderate", and 9 to 12 as "severe" [19]. For depressive symptoms alone (PHQ-2), scores from 0 to 2 were coded as "normal range", while scores from 3 to 6 were as "depressive symptoms" [20]. For Anxiety (GAD-2), sum scores from 0 to 2 were coded as "normal range", while scores from 3 to 6 were coded as "anxiety symptoms" [21].

#### Quality of life

Participants perceived quality of life was measured using the EUROHIS-QOL 8-item Index [22]. The self-assessment questionnaire was developed as an adaptation of the WHOQOL-100 and the WHOQOL-BREF instruments [23]. It includes items evaluating life satisfaction, satisfaction with own health, abilities to get things done, and satisfaction with self and personal relationships. Questions can be answered on a Likert Scale ranging from 1 to 5, with sum scores ranging from 8 to 40. Higher scores indicate better quality of life. The EUROHIS-QOL 8 item presented good reliability in the current sample with a Cronbach's alpha value of 0.78.

#### Sociodemographic and socioeconomic characteristics

Participants were asked for their age ("how old are you?"), their gender (female, male, diverse), marital status (single/alone, married, in partnership, divorced, separated and widowed), and if they have children. In addition, participants were asked if they had left Ukraine with all family members or alone and how long they had been in Germany. Questions regarding the socioeconomic data were collected in terms of last occupation and highest educational attainment.

### Statistical analyses

Statistical analyses were computed using IBM SPSS Version 22. Descriptive statistics are reported as means ± standard deviations (SD), absolute frequencies and percentages. Sum scores were calculated to evaluate the general health (GHQ-12) and depression and anxiety symptoms (PHQ-4), respectively. T-test was used to check for possible significant differences between women and men. Multiple regression analysis was used to analyse potential associations between general health (GHQ-12) and depressive symptoms and anxiety (PHQ-4), and quality of life (EUROHIS-QOL 8 item).

## Results

As presented in Table 1 above, 235 of the total participants (n = 304) were female. Further descriptive analysis of sociodemographics confirms an average age of 40 for females and 37 for male participants. Most participants reported being in a partnership or married (61.8% of women and 74.2% of men). About 71% of female participants have at least one child. Similarly, 77.4% of the sample reported leaving close family member(s) back in Ukraine. Data on educational attainment shows that most participants have completed at least tertiary education (university bachelor's and master's degrees: 66.4% of women and 65.2% of men).

**Table 1:** Sociodemographic and socioeconomic characteristics of participants

	Male (n = 69)		Female (n = 235)		Total Sample (n = 304)	
	n	%	n	%	n	%
*Marital status*						
Single	13	18.8	36	15.3	49	16.1
Married	28	40.6	105	44.7	133	43.8
In partnership	23	33.6	40	17.1	63	20.7
Divorced	2	2.9	27	11.5	29	9.5
Widowed	1	1.4	16	6.8	17	5.6
Not specified	2	2.9	11	4.7	13	4.3
*Having children*						
Yes	35	50.7	167	71.1	202	66.4
No	34	49.3	68	28.9	102	33.6
*Arrived in Germany with all family members*						
Yes	24	34.8	53	22.6	77	25.3
No	45	65.2	182	77.4	227	74.7
*Highest educational attainment*						
None	–	–	3	1.3	3	1.0
Primary school	–	–	1	0.4	1	0.3
Secondary school	6	8.7	14	6.0	20	6.6
Technical college	5	7.2	34	14.5	39	12.8
Secondary school with University qualification	12	17.4	20	8.5	32	10.5
University bachelor's degree	31	44.9	61	26.0	92	30.3
University master's degree	14	20.3	95	40.4	109	35.9
University PhD or equivalent	–	–	4	1.7	4	1.3
Not specified	1	1.4	3	1.3	4	1.3

Average participant's age was 39 years old (SD = 11.84). For male participants, the average age was 37 years (SD = 10.58), while for females, the average score was 40 years (SD = 12.15)

As presented in Table 2, almost half of the female participants reported severe psychological distress (46.4%) compared to 20% of the male participants. Similarly, data on depressive symptoms and anxiety projects that 46.4% of men and 43.4% of women reported mild symptoms. On the other hand, about 45% of female and 26% of male participants reported moderate to severe symptoms. Similarly, separately aggregated scores for depressive symptoms and anxiety symptoms confirm that female participants reported higher scores in both outcomes.

**Table 2:** Mental health outcomes, percentage and frequency distribution by gender and for the total sample

	Total Sample (n = 304)		Male (n = 69)		Female (n = 235)		Chi-Square Tests Gender diff	
	n	%	n	%	n	%	X^2^	*p*
General psychological distress (GHQ-12)							**31.56**	**.000**
Normal range	78	25.7	35	50.7	43	18.3		
Mild distress	34	11.2	6	8.7	28	11.9		
Distress	69	22.7	14	20.3	55	23.4		
Severe psychological distress	123	40.5	14	20.3	109	46.4		
Depressive symptoms and anxiety (PHQ-4)							**13.88**	**.003**
Normal	47	15.5	19	27.5	28	11.9		
Mild	134	44.1	32	46.4	102	43.4		
Moderate	61	20.1	11	15.9	50	21.3		
Severe	62	20.4	7	10.1	55	23.4		
Depressive Symptoms (PHQ-2)							**4.6949**	**.030**
Normal range	168	55.3	46	66.7	122	51.9		
Depressive symptoms	136	44.7	23	33.3	113	48.1		
Anxiety (GAD-2)							0.7591	.383
Normal range	149	49.0	37	53.6	112	47.7		
Anxiety symptoms	155	51.0	32	46.4	123	52.3		

Bold highlights significant differences based on gender

A chi-square test of independence was performed to examine the relationship between gender and general psychological distress. The results confirm a significant difference, *X*^2^ (3, N = 304) = 31.56, *p* = .000, suggesting that females were likelier than males to report psychological distress. Similar analysis on depressive symptoms and anxiety also returns significant results *X*^*2*^ (3, N = 304) = 13.88, *p* = .003, which puts female participants at a disadvantage for depressive symptoms and anxiety. A separate chi-square test for depressive symptoms and gender suggests a significant difference favouring male *X*^*2*^ (3, N = 304) = 4.70, *p* = .030. However, the chi-square test for anxiety showed no significant association with gender, *X*^2^ (3, *N* = 304) = 0.76, *p* = .383.

### Statistical differences in mental health outcomes and quality of life based on gender

The independent t-test shows statistically significant differences in mental health outcomes for male and female participants (see. Table 3). Female participants reported significantly higher psychological distress as measured by the GHQ-12. Similarly, as well as depressive symptoms and anxiety (PHQ-4). On the other hand, no significant statistical difference was found for the quality of life score by gender (EUROHIS-QOL 8 item).

**Table 3:** Independent t-test for gender differences in mental health and quality of life outcome

	Male		Female		Cohen's D	t	Sig. (2-tailed)
	M	SD	M	SD			
Quality of life (EUROHIS-QOL 8)	23.78	5.59	22.47	4.75	0.26	1.937	.054
Psychological distress (GHQ-12)	14.20	6.55	18.90	6.64	− 0.68	− 5.186	**.000**
Depressive symptoms and anxiety (PHQ-4)	4.39	2.80	5.85	3.15	− 0.46	− 3.688	**.000**
Depressive symptom (PHQ-2)	2.03	1.52	2.89	1.75	− 0.49	− 3.962	**.000**
Anxiety symptom (GAD -2)	2.36	1.62	2.96	1.75	− 0.35	− 2.549	**.011**

Bold highlights a significant different between males and females at *p* ≤ 0.05

### Associations between quality of life and mental health outcomes

Spearman's rho correlation shows positive associations between quality of life and mental health outcomes (see Table 4). Higher quality of life score was strongly associated with lower psychological distress (r = − 0.562, *p* < 0.01). Similar results were reported for the associations between quality of life and depressive symptoms (r = − 0.562, *p* < 0.01) and anxiety (r = − 0.562, *p* < 0.01). Age returns no statistical significance for the quality of life or mental health outcomes. However, moderate to weak associations were found between gender and mental health outcomes.

**Table 4:** Spearman's rho Correlation Matrix for samples' psychological health and quality of life outcome (n = 304)

		M	SD	1	2	3	4	5	6	7
1	Quality of life (EUROHIS-QOL)	22.8	5.0	1						
2	General psychological distress (GHQ-12)	17.8	6.9	− .562**	1					
3	Depressive symptoms and anxiety (PHQ-4)	5.5	3.1	− .543**	.657**	1				
4	Depressive symptoms (PHQ-2)	2.7	1.7	− .478**	.592**	.886**	1			
5	Anxiety (GAD-2)	2.8	1.7	− .508**	.591**	.905**	.623**	1		
6	Age	39.1	11.8	− .003	− .005	− .086	− .093	− .064	1	
7	Gender	1.8	0.4	− .090	.295**	.184**	.199**	.133*	.089	1

**Correlation is significant at the 0.01 level (2-tailed). *Correlation is significant at the 0.05 level (2-tailed)

Means, standard deviations and correlations between quality of life, psychological health outcomes, age and gender are presented in Table 4. All psychological health outcomes negatively correlate with the quality of life. Age has no significant correlation with psychological health outcomes or quality of life. Gender positively correlates with all psychological health outcomes but not with the quality of life.

Multiple linear regression was calculated to test mental health outcomes as a predictor of quality of life separately for males, females, and the total sample. The significant model (*p* < .001) for the males accounts for 33.6% of the variance in quality of life. General psychological distress (β = − .240) and depressive symptoms and anxiety (β = − .411) are associated with decreased quality of life. For the female sample (p < .001), the model explaines 35.7% of the variance in quality of life. General psychological distress (β = − .402) and depressive symptoms and anxiety (β = − .261) are associated with decreased quality of life (Table 5).

**Table 5:** Multiple linear regression for psychological health outcomes as predictors of quality of life by gender and for the total sample

	Model 1—male (n = 69)			Model 2—female (n = 235)			Model 3—total sample (n = 304)		
	B	β	*p*	B	β	*p*	B	β	*p*
Constant	30.293		< .001	30.197		< .001	30.020		< .001
General psychological distress (GHQ-12)	− .205	− .240	.070	− .287	− .402	< .001	− .262	− .364	< .001
Depressive symptoms and anxiety (PHQ-4)	− .821	− .411	.002	− .393	− .261	< .001	− .468	− .294	< .001
*Model fit indices*									
Adjusted R^2^	.336			.357			.355		
ΔF (df1, df2), *p*-value	18.238 (2, 66), *p* < 0.001			65.843 (2, 232), *p* < 0.001			85.231 (2, 301), *p* < 0.001		

## Discussion

Since the onset of the war in February 2022, more Ukrainians have continued to seek refuge in Germany. The current research examines the mental health outcomes, gender differences and the association with quality of life among a sample of Ukrainian refugees in Germany.

The current results suggest a high prevalence of mental health problems measured by general psychological distress, depressive symptoms, and anxiety among the Ukraine refugee sample. More than 60% of the participants reported substantial or severe psychological distress attributed to loss of sleep over worry, constant feeling under strain, and losing confidence or self-worth, among others. Similarly, nearly half of the participants reported depressive symptoms, while over half reported anxiety symptoms concerning being nervous, anxious, or tense and unable to stop or control worrying. Previous research reports varying prevalences of mental health problems among refugees [8, 24].

While the variation or high prevalence of mental health problems among refugees is usually attributed to premigration and migration factors, new evidence has highlighted post-migrational factors, for example, integration, as predictors of refugee mental health outcomes [25, 26]. For instance, the study by Walther et al. [26] shows the influence of integration on mental health. Here, it is concluded that fundamental tasks during the integration process, such as bureaucratic obstacles and social disconnection, can negatively affect mental health. For the current sample, however, we argue that while the positive reception and various legal frameworks to facilitate the integration of Ukrainian refugees in Germany might have reduced the burden of mental health problems among this refugee group, the deeming effect of the war they flee significantly affect their mental health outcomes. Compared to refugees from Syria, Iraq or Afghanistan, Ukrainian refugees in Germany experienced and are still experiencing far-reaching benefits regarding admission and social benefits. Nevertheless, their mental health outcomes can be attributed to uncertainties related to the war in their home country. The war and the associated experiences pre- and post-migration overshadowed the "welcome structured advantages". Regardless of the treatment in the host country, the effect of war is evident in the Ukrainian refugee's mental health outcomes.

The second objective of this study was to explore possible gender differences in mental health outcomes and quality of life. The results show significant differences in mental health outcomes based on gender but no differences in quality of life. Female participants reported poorer mental health outcomes compared to male participants. This finding collaborates with other research findings suggesting that female migrants and refugees are disadvantaged in mental health compared to males [27–29]. Hollander et al. [28], for example, found in a sample of 43,168 refugees and non-refugees, of whom 20,940 (48.5%) were women and 24,403 (56.5%) were refugees, that being a female refugee is a risk factor for mental health problems. They found a significant difference in the odds ratio for refugee and non-refugee women not explained by age, origin, marital status, and education. In other words, female refugees are more likely to be stressed, depressed and show anxiety symptoms. For the current sample, these gender differences are further explained by Ukraine's political situation. For example, men can only leave Ukraine and seek refuge elsewhere due to disability, old age or other clearly stated exceptions [16]. Male Ukrainian refugees in Germany may feel fortunate to have left Ukraine, which may boost their mental health.

Another explanation for the poorer mental health of females may be separation from the family. About 77.4% of the female participants had left close family members back in Ukraine and may be worried about their safety. This is especially so since many of the men left back home are in the army on the front lines, where their lives are directly threatened. Furthermore, classic role models that place men as family providers are predominant in Ukraine [30]. Men usually have higher socioeconomic status, for example, better employment and higher income. For the female sample, the absence of the support they received can mean an additional burden.

Similarly, the socialisation of the image of women and men in Ukraine is somewhat more conservative, and especially from men, strength is expected. Therefore, we assume that answers from men have been given in the sense of social desirability. Thus, fewer mental health problems may be reported by the male participants.

The third objective was to explore mental health outcomes as predictors of quality of life. Results from multiple linear regression confirm that both psychological distress and depressive symptoms, and anxiety significantly negatively impact the female participants' quality of life. This means that the quality of life decreases with increased psychological stress or/and depressive symptoms and anxiety. For male participants, depressive symptoms and anxiety significantly predict the quality of life. These results are similar to Leiert et al. [31], where all health outcome measures negatively correlate with the self-assessed quality of life among 510 refugees living in Sweden. The authors found the strongest association between depression and perceived mental health. Similarly, Belau et al. [32] find that social integration, social support, and loneliness correlate with the quality of life within the group of 326 refugees living in North Rhine-Westphalia, Germany. The computed variances of 33.6% (men) and 35.7% (women) are relatively high and suggest the increased importance of mental health as a crucial predictor of quality of life among this refugee group. Dangman et al. [33] found that potentially traumatic events from war reduce the quality of life and that this was mediated by post-migration stressors alone or in sequence with mental distress.

### Limitations

It is important to take note of the following limitations while interpreting the results presented here. First, the cross-sectional nature of this study limits the generalisability of the findings. It can be assumed that mental health and quality of life change with the duration of stay and the stage of integration of the participants. A longitudinal study will provide a more in-depth view of how mental health outcomes predict the Ukrainian refugees' quality of life in Germany. Secondly, the current analysis uses a self-reported measure of mental health outcomes and quality of life. It cannot be ruled out that answers have been given in the context of social desirability so that information bias may occur. Similarly, the current study used a self-translated version of the scales. Replicating the result in other Ukrainian samples is therefore crucial to establish the measure's clarity.

The sample used in the current analysis does not represent the total number of Ukrainian refugees in Germany. However, different approaches had to be used, as the recruitment of the target group proved complex and lengthy. Further studies using a more random and representative sampling technique will provide a more representative result on the Ukrainian refugee group's quality of life and mental health.

## Conclusion and implication for research and practices

Overall, far too little is known about the health of refugees from Ukraine. The current study provides the first knowledge on the prevalence of mental health problems and their associations with quality of life. The findings further identified the vulnerability of women refugees to poorer mental health outcomes. The results also confirm that traumatic experiences in the context of war explain a considerable bulk of mental health problems this refugee group faces. These results are crucial to establishing the first approach to providing care and understanding the health care need of this group. Furthermore, the current exploration offers a base for further and more comprehensive research to understand the underline factors that influence the mental health outcome of refugees in different contexts and settings.

Future research should consider a more robust sampling technique that produces more representative results. Our experience signifies the importance of building trust with participants by guaranteeing their anonymity. Furthermore, more research accounting for the socioeconomic status before arriving in Germany might provide deeper insight into how the social and political advantage of the Ukrainian refugee affected their mental health outcomes. Similarly, a comparison with other refugee groups would also reveal the peculiarity of this refugee group and the effectiveness of government policies and social actions to facilitate their integration into Germany. Qualitative research exploring the health and wellbeing of Ukrainian refugees will provide broader insight into the determinants of mental health and quality of life outcomes.

For policy and practices, past experiences have suggested that waiting too long for recommendations endangers the integration process [34]. Based on the current findings, actions and policies that promote mental health by facilitating sleep, self-confidence, and concentration and addressing depressive symptoms and anxiety will significantly improve the quality of life. Intervention projects tailored to meet specific gender needs will have a more positive effect on the mental health outcome of the Ukrainian refugees. Traumatic experiences in the context of war that lead to mental health problems must be addressed adequately.

## Data Availability

The data supporting this study's findings are available from the corresponding author, Johanna Buchcik, upon request.
